# Supplementary material for: Foraging of Honeybees from Different Ecological Areas Determined through Melissopalynological Analysis and DNA Metabarcoding
Source: Insects. 2024 Sep 5;15(9):674. doi: 10.3390/insects15090674 (PMC11432334; doi:10.3390/insects15090674)
Supplement: Supplementary file 1 [file insects-15-00674-s001.zip › Table S1.pdf]

Table S1. The most common plant taxa determined by melisopalynology.

Family	Species	Number of pollen types	Percent
**intensive farming area**			
Polygonaceae	*Fagopyrum esculentum Moench*	571	53%
Astraceae (Compositae)	*Helianthus annuus L.*	186	17%
Urticaceae	*Urtica dioica L.*	75	7%
Gentianaceae	*Centaurium erythraea Rafin*	67	6%
Fabacea (Legumenosae)	*Lathyrus pratensis L.*	36	3%
Rosaceae	*Filipendula vulgaris Moench*	35	3%
Brassicaceae (Cruciferae)	*Brassica napus L.*	35	3%
Rubiaceae	*Galium mollugo L.*	33	3%
Ranunculaceae	*Ranunculus repens L*	26	2%
Apiaceae (Umbelliferae)	*Sium latifolum L*	20	2%
		**1084**	**100%**
**reserved area**			
Astraceae (Compositae)	*Helianthus annuus L.*	198	24%
Polygonaceae	*Fagopyrum esculentum Moench*	139	17%
Boraginaceae	*Echium vulagare L.*	138	17%
Brassicaceae (Cruciferae)	*Brassica napus L*	134	16%
Fabaceae (Legumenosae)	*Onobrychis arenaria(Kit.) DC.*	118	14%
Fabaceae (Legumenosae)	*Melilotus albus Medik.*	59	7%
Brassicaceae (Cruciferae)	*Sinapis alba L.*	24	3%
Fabaceae (Legumenosae)	*Melilotus officinalis(L.) Pall.*	6	1%
		**816**	100%
**urbanized area**			
Fabaceae (Legumenosae)	*Onobrychis arenaria(Kit.) DC.*	125	19%
Brassicaceae (Cruciferae)	*Brassica napus L*	108	17%
Fabaceae (Legumenosae)	*Melilotus albus Medik.*	82	13%
Brassicaceae (Cruciferae)	*Erysimum cheiranthoides L.*	81	12%
Boraginaceae	*Echium vulagare L*	71	11%
Astraceae (Compositae)	*Helianthus annuus L.*	62	10%
Fabaceae (Legumenosae)	*Ficaria verna Huds.*	43	7%
Fabaceae (Legumenosae)	*Melilotus officnalis (L.) Pall.*	34	5%

Salicacea	*Salix caprea L.*	23	4%
Fabaceae (Legumenosae)	*Trifolium canescens Willd.*	20	3%
		**649**	100%
