# Supplementary material for: Foraging of Honeybees from Different Ecological Areas Determined through Melissopalynological Analysis and DNA Metabarcoding
Source: Insects. 2024 Sep 5;15(9):674. doi: 10.3390/insects15090674 (PMC11432334; doi:10.3390/insects15090674)
Supplement: Supplementary file 1 [file insects-15-00674-s001.zip › Table S2.pdf]

Table S2. Plant taxa at the species level identified using DNA metabarcoding with *ITS2* and *rbcL* markers.

Family	Genus	Species	*ITS2*		*rbcL*	
			Abundance	Percent	Abundance	Percent процент
**intensive farming area**						
Brassicaceae	*Sinapis*	***Sinapis alba***	290772	**72%**	39403	3%
Brassicaceae	*Brassica*	*Brassica rapa subsp. oleifera*	0	0	118386	9%
Brassicaceae	*Brassica*	*Brassica nigra*	6533	2	21090	2%
Asteraceae	*Picris*	*Picris squarrosa*	32956	8%	0	0
Asteraceae	*Picris*	*Picris hieracioides*	0	0	166527	13%
Asteraceae	*Cirsium*	*Cirsium arvense*	2762	1%	0	0
Asteraceae	*Symphyotrichum*	*Symphyotrichum puniceum*	0	0	13162	1%
Asteraceae	*Helianthus*	***Helianthus divaricatus***	0	0	258525	**20%**
Polygonaceae	*Fagopyrum*	***Fagopyrum esculentum***	26895	6%	209950	**16%**
Fabaceae	*Trifolium*	*Trifolium repens*	15467	4%	49526	4%
Fabaceae	*Vicia*	*Vicia cracca*	12323	3%	0	0
Fabaceae	*Melilotus*	*Melilotus officinalis*	3627	1%	0	0
Fabaceae	*Melilotus*	*Melilotus albus*	0	0	86390	7%
Fabaceae	*Astragalus*	*Astragalus adsurgens*	0	0	21562	2%
Rosaceae	*Sanguisorba;*	*Sanguisorba officinalis*	11087	3%		0
Chenopodiaceae	*Bassia*	*Bassia scoparia*	4537	1%	63741	5%
Boraginaceae	*Nonea*	*Nonea caspica*	2632	1%	0	0
Plantaginaceae	*Linaria*	*Linaria vulgaris*	0	0	145455	11%
Orobanchaceae	*Odontites*	*Odontites hispidulus*	0	0	28947	2%
Lythraceae	*Lythrum*	*Lythrum salicaria*	0	0	16873	1%
Menyanthaceae	*Nymphoides*	*Nymphoides peltata*	0	0	15236	1%
Cannabaceae	*Cannabis*	*Cannabis sativa*	0	0	13847	1%
Cucurbitaceae	*Cucumis*	*Cucumis hystrix*	0	0	13462	1%
Convolvulaceae	*Convolvulus*	*Convolvulus arvensis*	0	0	11827	1%
			**409591**	**100**	**1293909**	**100**
**reserved area**						
Rosaceae	*Filipendula*	***Filipendula vulgaris***	7410	**20%**	10184	2%
Rosaceae	*Sanguisorba;*	*Sanguisorba officinalis*	1476	4%	0	0
Boraginaceae	*Echium*	***Echium vulgare***	6216	**17%**	0	0
Fabaceae	*Melilotus*	***Melilotus officinalis***	5817	**16%**	0	0
Fabaceae	*Melilotus*	***Melilotus albus***	0	0	132572	**20%**
Fabaceae	*Caragana*	*Caragana arborescens*	0	0	10913	2%

Fabaceae	*Onobrychis*	*Onobrychis viciifolia*	0	0	10008	1%
Convolvulaceae	*Convolvulus*	*Convolvulus arvensis*	4361	12%	14966	2%
Polygonaceae	*Fagopyrum*	***Fagopyrum esculentum***	2611	7%	0	0
Brassicaceae	*Raphanus*	*Raphanus sativus*	1898	5%	0	0
Brassicaceae	*Sisymbrium*	*Sisymbrium loeselii*	1487	4%	0	0
Brassicaceae	*Brassica*	*Brassica napus*	1437	4%	0	0
Brassicaceae	*Brassica*	*Brassica rapa subsp. oleifera*	0	0	106276	16%
Lamiaceae	*Hyssopus*	*Hyssopus officinalis*	1782	3%	0	0
Solanaceae	*Petunia*	*Unknown Petunia*	1528	4%	0	0
Solanaceae	*Solanum*	*Solanum lycopersicum*	1397	4%	0	0
Solanaceae	*Solanum*	*Solanum cheesmaniae*	0	0	13966	3%
Asteraceae	*Helianthus*	***Helianthus divaricatus***	0	0	156199	**24%**
Asteraceae	*Solidago*	*Solidago rugosa*	0	0	82305	12%
Asteraceae	*Centaurea*	*Centaurea nigra*	0	0	29006	4%
Asteraceae	*Tripolium*	*Tripolium pannonicum*	0	0	9257	1%
Geraniaceae	*Erodium*	*Erodium carvifolium*	0	0	47344	7%
Poaceae	*Triticum*	*Triticum aestivum*	0	0	19857	4%
Caprifoliaceae	*Lonicera*	*Lonicera tatarica*	0	0	15205	2%
			**37420**	**100%**	**658058**	**100%**
**urbanized area**						
Brassicaceae;	*Raphanus;*	***Raphanus sativus***	35763	**31%**	0	0
Brassicaceae;	*Diplotaxis;*	*Diplotaxis harra*	4645	4%	0	0
Fabaceae;	*Trifolium;*	***Trifolium pratense***	18732	**16%**	17977	3%
Fabaceae;	*Vicia;*	*Vicia cracca*	12654	11%	0	0
Fabaceae;	*Glycine;*	*Glycine max*	6303	5%	0	0
Fabaceae;	*Melilotus;*	*Melilotus officinalis*	3547	3%	0	0
Fabaceae	*Melilotus*	*Melilotus albus*	0	0	65985	10%
Fabaceae;	*Alhagi;*	*Alhagi maurorum*	3309	3%	0	0
Fabaceae	*Onobrychis*	*Onobrychis viciifolia*	0	0	13736	2%
Fabaceae	*Medicago*	*Medicago hybrida*	0	0	13709	2%
Asteraceae;	*Karelinia;*	*Karelinia caspia*	9292	8%	0	0
Asteraceae;	*Saussurea;*	*Saussurea elegans*	3472	3%	0	0
Asteraceae;	*Centaurea;*	*Centaurea depressa*	2306	3%	0	0
Asteraceae	*Helianthus*	*Helianthus divaricatus*	0	0	56355	9%
Asteraceae	*Centaurea*	*Centaurea nigra*	0	0	27247	4%
Boraginaceae;	*Echium;*	*Echium vulgare*	3039	3%	0	0
Onagraceae;;	*Chamaenerion*	*Chamaenerion angustifolium*	2641	2%	18375	3%
Rosaceae;	*Potentilla;*	*Potentilla agrimonioides*	2505	2%	0	0
Rosaceae;	*Spiraea;*	*Spiraea chartacea*	2289	2%	0	0

Convolvulaceae;	*Convolvulus;*	*Convolvulus arvensis*	2406	2%	17295	3%
Balsaminaceae;	*Impatiens;*	*Impatiens parviflora*	2147	2%	0	0
Cucurbitaceae	*Cucumis*	***Cucumis melo subsp. melo***	0	0	95702	**15%**
Cucurbitaceae	*Cucumis*	*Cucumis hystrix*	0	0	56400	9%
Malvaceae	*Malva*	*Malva dendromorpha*	0	0	25466	3%
Malvaceae	*Malva*	*Malva alcea*	0	0	30452	5%
Malvaceae	*Gossypium*	*Gossypium hirsutum*	0	0	51833	8%
Nitrariaceae	*Peganum*	*Peganum harmala*	0	0	35671	5%
Nitrariaceae	*Nitraria*	*Nitraria sphaerocarpa*	0	0	16692	3%
Amaranthaceae	*Oxybasis*	*Oxybasis glauca*	0	0	24420	3%
Poaceae	*Triticum*	*Triticum aestivum*	0	0	19228	2%
Poaceae	*Miscanthus*	*Miscanthus sacchariflorus*	0	0	11414	2%
Bignoniaceae	*Campsis*	*Campsis radicans*	0	0	18905	3%
Lamiaceae	*Phlomis*	*Phlomis jeholensis*	0	0	14944	2%
Lamiaceae	*Origanum*	*Origanum vulgare subsp. vulgare*	0	0	10937	2%
Capparaceae	*Capparis*	*Capparis spinosa*	0	0	10645	2%
			**115050**	**100%**	**653388**	100%
